# Vorticity for the assessment of right ventricular diastolic dysfunction using 4D flow CMR

**DOI:** 10.1186/1532-429X-15-S1-P8

**Published:** 2013-01-30

**Authors:** Brett Fenster, Jamey Browning, Aurelien F Stalder, Christopher Glielmi, Lori Silveira, J Kern Buckner, Alex Kluiber, Joyce D Schroeder, Jean Hertzberg

**Affiliations:** 1 Division of Cardiology, National Jewish Health, Denver, CO, USA; 2 Mechanical Engineering, University of Colorado, Boulder, CO, USA; 3 Division of Radiology, National Jewish Health, Denver, CO, USA; 4 Division of Biostatistics, National Jewish Health, Denver, CO, USA; 5 Siemens Healthcare, New York, NY, USA; 6 Magnetic Resonance, Imaging and Therapy, Healthcare Sector, Siemens AG, Erlangen, Germany

## Background

**Figure 1:**
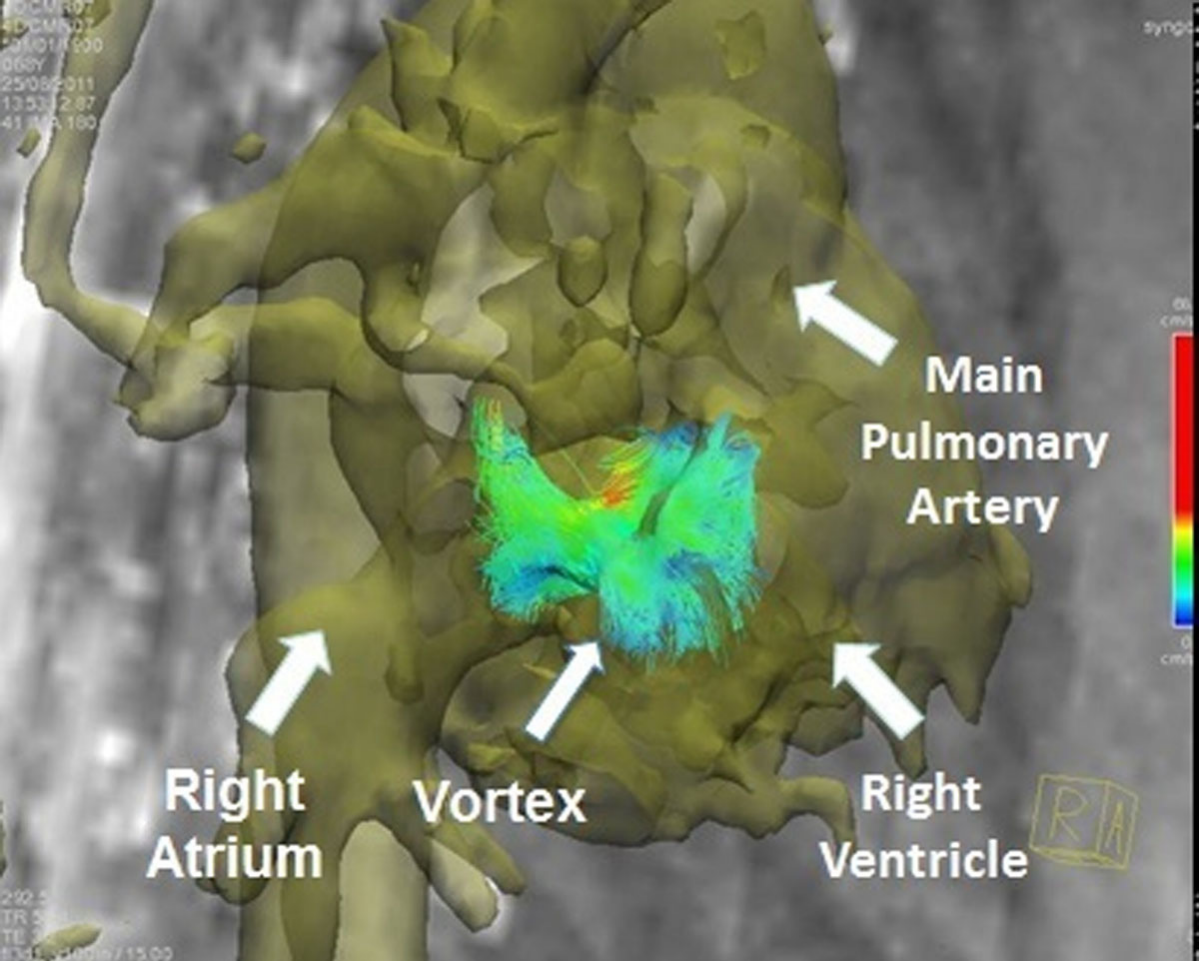
4D CMR image of RV diastolic inflow with anatomic masking in a control subject. Image was taken in right anterior oblique view demonstrating vortex formation at the edge of an open tricuspid valve.

4D flow CMR analysis of right ventricular (RV) diastolic inflow has demonstrated vortical formations at tips of the tricuspid valve during the deceleration phase of early filling (E wave) (Figure 1). Vorticity can measure the rotation of these vortices and may represent a novel way to assess RV diastolic function. We aimed to determine if right heart vorticity identified right ventricular diastolic dysfunction (RVDD) in pulmonary hypertension (PH) subjects when compared to controls using 4D flow CMR.

## Methods

Thirteen subjects (10 females/3 males) with PH and 10 age-matched controls (7 females/3 males) underwent same-day echocardiography and 4D flow CMR. Echocardiography assessed RV diastolic function and RV systolic pressure (RVSP). PH subjects had to demonstrate RVDD on the day of CMR as defined by either Stage I RVDD (tricuspid E/A < .8, E/E' < 6, and deceleration time (DT) > 120 ms) or Stage II RVDD (E/A > 2.1, E/E' of > or =6, or DT > 120 ms). 4D flow CMR was performed with interleaved 3-directional velocity encoding (spatial resolution=3.5×2.6×3.0 mm^3^, α=15°, TE/TR=2.85/48.56 ms, venc=150 cm/s, temporal resolution=50 ms) on a 1.5 T MRI system (Avanto, Siemens, Germany) using ECG gating and respiratory navigation. Images were acquired in a sagittal oblique 3D volume covering the entire right heart. Datasets were corrected for noise and aliasing using a custom Matlab program (Jelena Bock, Northwestern University) and imported into Paraview (Kitware, Clifton, NY) for isolation of the right heart field of view. Right heart E wave vorticity was calculated using Paraview. Univariate and multivariate regression analysis were used to test the relationship between E wave vorticity and RVDD using JMP (SAS, Cary, NC). Heart rate and cardiac index were used to control for RV filling time and RV inflow, respectively.

## Results

No significant difference in age or gender existed between control and RVDD groups (Table 1). RVSP was significantly elevated in RVDD vs. controls. All controls had normal RV diastolic function, while 6 PH subjects had Stage I RVDD and 7 had Stage II RVDD. Mean E wave vorticity was significantly (p 0.006) decreased in RVDD subjects (0.31+/-0.1 1/s) vs. controls (0.47+/-0.17 1/s). Decreased E wave vorticity correlated with RVDD presence (r 0.66, p 0.02) and severity (r 0.59, p 0.001) when controlled for age, gender, filling time, and RV inflow. E wave vorticity correlated with RVDD presence (r 0.51, p 0.01) and severity (r 0.37, p 0.008) remained significant even in the absence of filling time and RV inflow.

**Table 1:** Patient cohort characteristics

	Control	RVDD	p Value
Age (years)	57 +/- 12	63 +/- 6	NS
Gender (% Female)	70%	77%	NS
RVSP (mmHg)	58 +/- 17 mmHg	26 +/- 8 mmHg	p < .001

NS = not significant

## Conclusions

Decreased right heart vorticity identifies the presence and severity of RVDD in a small cohort of PH subjects when compared to controls. Vorticity analysis for the assessment of RVDD may represent a novel clinical application of 4D flow CMR. Future investigations will require improvements in 4D flow CMR spatial and temporal resolution, larger patient cohorts, and simultaneous 4D flow CMR and echocardiographic acquisition to validate this finding.

## Funding

Siemens Medical Solutions

